# 2-(4-Chloro­phen­yl)-1-phenyl-1*H*-benz­imidazole

**DOI:** 10.1107/S1600536812006678

**Published:** 2012-02-24

**Authors:** Karimah Kassim, N. Zakiah N. Hashim, Adibatul Husna Fadzil, M. Sukeri M. Yusof

**Affiliations:** a Department of Chemistry, Faculty of Applied Sciences, Universiti Teknologi MARA, 40450 Shah Alam, Selangor, Malaysia; b Department of Chemical Sciences, Faculty of Science and Technology, Universiti Malaysia Terengganu, 21030 Kuala Terengganu, Terengganu, Malaysia

## Abstract

In the title compound, C_19_H_13_ClN_2_, the dihedral angle formed by the imidazole core with the chloro­phenyl and phenyl rings are 24.07 (4) and 67.24 (4)°, respectively.

## Related literature

For the applications of benzimidazoles derivatives, see: Velík *et al.* (2004 ▶); Aljourani *et al.* (2009 ▶); Tiwari *et al.* (2007 ▶). For related structures, see: Nor Hashim *et al.* (2010 ▶); Arumugam *et al.* (2010 ▶). For standard bond lengths, see: Allen *et al.* (1987 ▶).
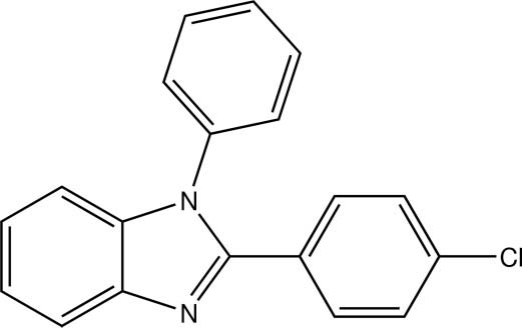


## Experimental

### 

#### Crystal data

C_19_H_13_ClN_2_
*M* *_r_* = 304.76
Monoclinic, 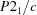

*a* = 8.2981 (1) Å
*b* = 9.2963 (2) Å
*c* = 20.7796 (3) Å
β = 112.815 (1)°
*V* = 1477.56 (4) Å^3^
*Z* = 4
Mo *K*α radiation
μ = 0.26 mm^−1^
*T* = 293 K
0.48 × 0.39 × 0.18 mm

#### Data collection

Bruker APEX DUO CCD area-detector diffractometer
Absorption correction: multi-scan (*SADABS*; Bruker, 2009 ▶) *T*_min_ = 0.887, *T*_max_ = 0.956
33103 measured reflections
5398 independent reflections
4610 reflections with *I* > 2σ(*I*)
*R*_int_ = 0.027

#### Refinement

*R*[*F* ^2^ > 2σ(*F* ^2^)] = 0.039
*wR*(*F* ^2^) = 0.106
*S* = 1.04
5398 reflections
199 parameters
H-atom parameters constrained
Δρ_max_ = 0.49 e Å^−3^
Δρ_min_ = −0.28 e Å^−3^

### 

Data collection: *APEX2* (Bruker, 2009 ▶); cell refinement: *SAINT* (Bruker, 2009 ▶); data reduction: *SAINT*; program(s) used to solve structure: *SHELXTL* (Sheldrick, 2008 ▶); program(s) used to refine structure: *SHELXTL*; molecular graphics: *SHELXTL*; software used to prepare material for publication: *SHELXTL*, *PARST* (Nardelli, 1995 ▶) and *PLATON* (Spek, 2009 ▶).

## Supplementary Material

Crystal structure: contains datablock(s) global, I. DOI: 10.1107/S1600536812006678/bh2413sup1.cif

Structure factors: contains datablock(s) I. DOI: 10.1107/S1600536812006678/bh2413Isup2.hkl

Supplementary material file. DOI: 10.1107/S1600536812006678/bh2413Isup3.cml

Additional supplementary materials:  crystallographic information; 3D view; checkCIF report

## Figures and Tables

**Fig. 1.**
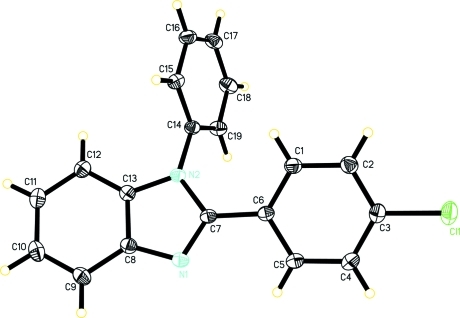
The molecular structure of the title compound, with displacement ellipsoids drawn at the 50% probability level.

**Fig. 2.**
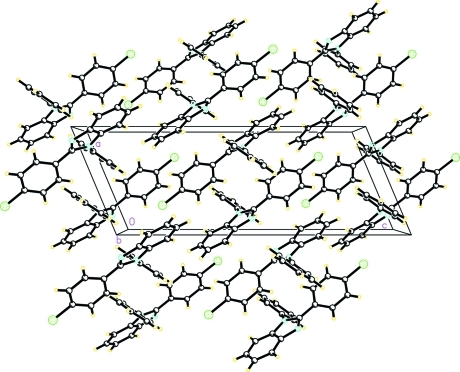
A packing diagram of the title compound viewed down the b axis.

## supplementary crystallographic information

### Comment

Benzimidazoles derivatives exhibit wide interest, especially in fields as biological compounds (Velík *et al.*, 2004), corrosion inhibitors (Aljourani *et al.*, 2009) and medicinal related chemistry (Tiwari *et al.*, 2007). A number of synthesis routes for substituted benzimidazole-containing structures have been developed, affording molecules that posses significant activity.

The title compound (Fig. 1) contains three six- and a one five-membered rings. The bond lengths and angles are within normal ranges (Allen *et al.*, 1987) and comparable to those found in *N*-[(*E*)-4-chlorobenzylidene]-*N'*-phenylbenzene-1,4-diamine (Nor Hashim *et al.*, 2010) and ethyl 1-*sec*-butyl-2-(2-hydroxyphenyl)-1*H*-benzimidazole-5-carboxylate (Arumugam *et al.*, 2010). The dihedral angle between benzene (C1···C6) and benzimidazole (N1/N2/C7···C13) rings is 24.07 (4)°. In the crystal structure (Fig. 2), there are no intra- and inter-molecule interactions.

### Experimental

4-Chlorobenzaldehyde (0.50 g, 3.6 mmol) in 10 ml of ethanol and *N*-phenyl-*o*-phenylenediamine (0.66 g, 3.6 mmol) in 10 ml of ethanol, were mixed in a round bottom flask. The mixture was refluxed for 5 h. The reaction mixture was then cooled to room temperature and left to stand in an open air vessel for about 48 h. Brown crystals were collected after evaporation of the solvent. Yield: 65%; m.p. 150.0–150.5°C.

### Refinement

C-bonded H atoms were positioned geometrically with C—H = 0.93 Å and constrained to ride on their parent atoms with *U*_iso_(H)= 1.2*U*_eq_(parent atom).

### Crystal data

**Table:**

C_19_H_13_ClN_2_	*F*(000) = 632
*M**_r_* = 304.76	*D*_x_ = 1.370 Mg m^−^^3^
Monoclinic, *P*2_1_/*c*	Melting point: 423 K
Hall symbol: -P 2ybc	Mo *K*α radiation, λ = 0.71073 Å
*a* = 8.2981 (1) Å	θ = 2.1–32.7°
*b* = 9.2963 (2) Å	µ = 0.26 mm^−^^1^
*c* = 20.7796 (3) Å	*T* = 293 K
β = 112.815 (1)°	Slab, brown
*V* = 1477.56 (4) Å^3^	0.48 × 0.39 × 0.18 mm
*Z* = 4	

### Data collection

**Table:**

Bruker APEX DUO CCD area-detector diffractometer	5398 independent reflections
Radiation source: fine-focus sealed tube	4610 reflections with *I* > 2σ(*I*)
Graphite monochromator	*R*_int_ = 0.027
Detector resolution: 83.66 pixels mm^-1^	θ_max_ = 32.7°, θ_min_ = 2.1°
ω scan	*h* = −9→12
Absorption correction: multi-scan (*SADABS*; Bruker, 2009)	*k* = −13→14
*T*_min_ = 0.887, *T*_max_ = 0.956	*l* = −31→31
33103 measured reflections	

### Refinement

**Table:**

Refinement on *F*^2^	Primary atom site location: structure-invariant direct methods
Least-squares matrix: full	Secondary atom site location: difference Fourier map
*R*[*F*^2^ > 2σ(*F*^2^)] = 0.039	Hydrogen site location: inferred from neighbouring sites
*wR*(*F*^2^) = 0.106	H-atom parameters constrained
*S* = 1.04	*w* = 1/[σ^2^(*F*_o_^2^) + (0.0532*P*)^2^ + 0.487*P*] where *P* = (*F*_o_^2^ + 2*F*_c_^2^)/3
5398 reflections	(Δ/σ)_max_ = 0.001
199 parameters	Δρ_max_ = 0.49 e Å^−^^3^
0 restraints	Δρ_min_ = −0.28 e Å^−^^3^
0 constraints	

### Fractional atomic coordinates and isotropic or equivalent isotropic displacement parameters (Å^2^)

**Table:**

	*x*	*y*	*z*	*U*_iso_*/*U*_eq_	
Cl1	0.72432 (4)	−0.07747 (3)	−0.201084 (13)	0.02762 (8)	
N1	0.15677 (11)	0.13941 (9)	−0.51877 (4)	0.01712 (15)	
N2	0.19862 (10)	−0.08273 (8)	−0.55312 (4)	0.01549 (15)	
C1	0.49136 (12)	−0.11517 (11)	−0.40861 (5)	0.01843 (17)	
H1B	0.5057	−0.1695	−0.4436	0.022*	
C2	0.60543 (13)	−0.13482 (11)	−0.33949 (5)	0.02010 (18)	
H2A	0.6952	−0.2021	−0.3281	0.024*	
C3	0.58327 (13)	−0.05256 (11)	−0.28778 (5)	0.01930 (18)	
C4	0.45136 (13)	0.05008 (11)	−0.30385 (5)	0.01991 (18)	
H4A	0.4393	0.1057	−0.2688	0.024*	
C5	0.33807 (13)	0.06833 (10)	−0.37285 (5)	0.01777 (17)	
H5A	0.2494	0.1366	−0.3840	0.021*	
C6	0.35547 (12)	−0.01485 (10)	−0.42611 (5)	0.01522 (16)	
C7	0.23567 (12)	0.01388 (10)	−0.49847 (5)	0.01518 (16)	
C8	0.06201 (12)	0.12524 (10)	−0.59018 (5)	0.01704 (17)	
C9	−0.04641 (13)	0.22408 (11)	−0.63856 (5)	0.02113 (19)	
H9A	−0.0658	0.3155	−0.6248	0.025*	
C10	−0.12370 (13)	0.18085 (12)	−0.70759 (6)	0.0235 (2)	
H10A	−0.1965	0.2445	−0.7406	0.028*	
C11	−0.09486 (14)	0.04291 (12)	−0.72905 (5)	0.0232 (2)	
H11A	−0.1474	0.0183	−0.7760	0.028*	
C12	0.00988 (13)	−0.05700 (11)	−0.68185 (5)	0.02010 (18)	
H12A	0.0277	−0.1488	−0.6956	0.024*	
C13	0.08723 (12)	−0.01221 (10)	−0.61242 (5)	0.01645 (16)	
C14	0.23702 (12)	−0.23309 (10)	−0.55279 (5)	0.01539 (16)	
C15	0.33740 (12)	−0.28012 (11)	−0.58889 (5)	0.01818 (17)	
H15A	0.3838	−0.2146	−0.6109	0.022*	
C16	0.36742 (13)	−0.42709 (11)	−0.59159 (5)	0.02123 (19)	
H16A	0.4343	−0.4601	−0.6155	0.025*	
C17	0.29783 (13)	−0.52407 (11)	−0.55872 (6)	0.0221 (2)	
H17A	0.3173	−0.6221	−0.5611	0.027*	
C18	0.19916 (14)	−0.47574 (11)	−0.52227 (6)	0.0226 (2)	
H18A	0.1546	−0.5413	−0.4996	0.027*	
C19	0.16673 (13)	−0.32917 (11)	−0.51960 (5)	0.01932 (18)	
H19A	0.0990	−0.2963	−0.4959	0.023*	

### Atomic displacement parameters (Å^2^)

**Table:**

	*U*^11^	*U*^22^	*U*^33^	*U*^12^	*U*^13^	*U*^23^
Cl1	0.03461 (15)	0.02418 (13)	0.01779 (12)	−0.00362 (10)	0.00329 (10)	0.00170 (8)
N1	0.0187 (3)	0.0134 (3)	0.0199 (4)	0.0005 (3)	0.0082 (3)	−0.0005 (3)
N2	0.0178 (3)	0.0120 (3)	0.0166 (3)	−0.0001 (3)	0.0067 (3)	−0.0015 (3)
C1	0.0184 (4)	0.0169 (4)	0.0200 (4)	0.0007 (3)	0.0075 (3)	−0.0019 (3)
C2	0.0195 (4)	0.0176 (4)	0.0213 (4)	0.0002 (3)	0.0060 (3)	−0.0005 (3)
C3	0.0219 (4)	0.0177 (4)	0.0170 (4)	−0.0043 (3)	0.0061 (3)	0.0005 (3)
C4	0.0244 (4)	0.0187 (4)	0.0185 (4)	−0.0033 (3)	0.0103 (3)	−0.0031 (3)
C5	0.0190 (4)	0.0155 (4)	0.0204 (4)	−0.0011 (3)	0.0094 (3)	−0.0024 (3)
C6	0.0160 (4)	0.0130 (4)	0.0176 (4)	−0.0020 (3)	0.0076 (3)	−0.0013 (3)
C7	0.0162 (4)	0.0130 (4)	0.0178 (4)	−0.0010 (3)	0.0082 (3)	−0.0018 (3)
C8	0.0174 (4)	0.0149 (4)	0.0195 (4)	−0.0005 (3)	0.0080 (3)	0.0009 (3)
C9	0.0217 (4)	0.0173 (4)	0.0248 (5)	0.0015 (3)	0.0096 (4)	0.0044 (3)
C10	0.0218 (4)	0.0256 (5)	0.0222 (5)	0.0014 (4)	0.0077 (4)	0.0076 (4)
C11	0.0222 (4)	0.0286 (5)	0.0178 (4)	−0.0015 (4)	0.0066 (3)	0.0018 (4)
C12	0.0201 (4)	0.0216 (4)	0.0186 (4)	−0.0015 (3)	0.0074 (3)	−0.0017 (3)
C13	0.0162 (4)	0.0158 (4)	0.0176 (4)	−0.0012 (3)	0.0069 (3)	0.0004 (3)
C14	0.0155 (4)	0.0129 (4)	0.0174 (4)	−0.0008 (3)	0.0060 (3)	−0.0022 (3)
C15	0.0180 (4)	0.0191 (4)	0.0184 (4)	−0.0008 (3)	0.0081 (3)	−0.0032 (3)
C16	0.0185 (4)	0.0220 (5)	0.0213 (4)	0.0025 (3)	0.0057 (3)	−0.0068 (3)
C17	0.0197 (4)	0.0149 (4)	0.0265 (5)	0.0015 (3)	0.0031 (4)	−0.0045 (3)
C18	0.0226 (4)	0.0143 (4)	0.0304 (5)	−0.0018 (3)	0.0098 (4)	0.0005 (4)
C19	0.0199 (4)	0.0159 (4)	0.0249 (5)	−0.0010 (3)	0.0117 (4)	−0.0005 (3)

### Geometric parameters (Å, º)

**Table:**

Cl1—C3	1.7418 (10)	C9—C10	1.3845 (15)
N1—C7	1.3245 (12)	C9—H9A	0.9300
N1—C8	1.3902 (12)	C10—C11	1.4083 (16)
N2—C13	1.3853 (12)	C10—H10A	0.9300
N2—C7	1.3856 (12)	C11—C12	1.3853 (15)
N2—C14	1.4331 (12)	C11—H11A	0.9300
C1—C2	1.3921 (14)	C12—C13	1.3960 (14)
C1—C6	1.3985 (13)	C12—H12A	0.9300
C1—H1B	0.9300	C14—C19	1.3876 (13)
C2—C3	1.3880 (14)	C14—C15	1.3900 (13)
C2—H2A	0.9300	C15—C16	1.3938 (14)
C3—C4	1.3917 (15)	C15—H15A	0.9300
C4—C5	1.3867 (14)	C16—C17	1.3852 (16)
C4—H4A	0.9300	C16—H16A	0.9300
C5—C6	1.4026 (13)	C17—C18	1.3880 (15)
C5—H5A	0.9300	C17—H17A	0.9300
C6—C7	1.4701 (13)	C18—C19	1.3942 (14)
C8—C9	1.4011 (13)	C18—H18A	0.9300
C8—C13	1.4019 (13)	C19—H19A	0.9300
			
C7—N1—C8	105.16 (8)	C9—C10—C11	121.67 (10)
C13—N2—C7	106.36 (8)	C9—C10—H10A	119.2
C13—N2—C14	122.43 (8)	C11—C10—H10A	119.2
C7—N2—C14	130.55 (8)	C12—C11—C10	121.55 (10)
C2—C1—C6	120.86 (9)	C12—C11—H11A	119.2
C2—C1—H1B	119.6	C10—C11—H11A	119.2
C6—C1—H1B	119.6	C11—C12—C13	116.27 (10)
C3—C2—C1	119.03 (9)	C11—C12—H12A	121.9
C3—C2—H2A	120.5	C13—C12—H12A	121.9
C1—C2—H2A	120.5	N2—C13—C12	131.22 (9)
C2—C3—C4	121.33 (9)	N2—C13—C8	105.76 (8)
C2—C3—Cl1	119.33 (8)	C12—C13—C8	123.03 (9)
C4—C3—Cl1	119.34 (8)	C19—C14—C15	121.41 (9)
C5—C4—C3	119.13 (9)	C19—C14—N2	119.64 (8)
C5—C4—H4A	120.4	C15—C14—N2	118.87 (8)
C3—C4—H4A	120.4	C14—C15—C16	118.94 (9)
C4—C5—C6	120.83 (9)	C14—C15—H15A	120.5
C4—C5—H5A	119.6	C16—C15—H15A	120.5
C6—C5—H5A	119.6	C17—C16—C15	120.19 (9)
C1—C6—C5	118.80 (9)	C17—C16—H16A	119.9
C1—C6—C7	122.98 (8)	C15—C16—H16A	119.9
C5—C6—C7	118.11 (8)	C16—C17—C18	120.35 (9)
N1—C7—N2	112.64 (8)	C16—C17—H17A	119.8
N1—C7—C6	122.71 (8)	C18—C17—H17A	119.8
N2—C7—C6	124.59 (8)	C17—C18—C19	120.12 (10)
N1—C8—C9	130.08 (9)	C17—C18—H18A	119.9
N1—C8—C13	110.08 (8)	C19—C18—H18A	119.9
C9—C8—C13	119.84 (9)	C14—C19—C18	118.98 (9)
C10—C9—C8	117.62 (10)	C14—C19—H19A	120.5
C10—C9—H9A	121.2	C18—C19—H19A	120.5
C8—C9—H9A	121.2		
			
C6—C1—C2—C3	−0.36 (15)	C9—C10—C11—C12	1.15 (16)
C1—C2—C3—C4	−0.95 (15)	C10—C11—C12—C13	−1.15 (15)
C1—C2—C3—Cl1	179.49 (8)	C7—N2—C13—C12	179.43 (10)
C2—C3—C4—C5	1.21 (15)	C14—N2—C13—C12	−9.02 (16)
Cl1—C3—C4—C5	−179.23 (7)	C7—N2—C13—C8	−0.15 (10)
C3—C4—C5—C6	−0.16 (15)	C14—N2—C13—C8	171.41 (8)
C2—C1—C6—C5	1.37 (14)	C11—C12—C13—N2	−179.23 (10)
C2—C1—C6—C7	177.63 (9)	C11—C12—C13—C8	0.28 (15)
C4—C5—C6—C1	−1.11 (14)	N1—C8—C13—N2	0.38 (10)
C4—C5—C6—C7	−177.54 (9)	C9—C8—C13—N2	−179.76 (8)
C8—N1—C7—N2	0.37 (10)	N1—C8—C13—C12	−179.24 (9)
C8—N1—C7—C6	177.69 (8)	C9—C8—C13—C12	0.62 (15)
C13—N2—C7—N1	−0.14 (10)	C13—N2—C14—C19	−107.24 (11)
C14—N2—C7—N1	−170.76 (9)	C7—N2—C14—C19	62.08 (13)
C13—N2—C7—C6	−177.40 (8)	C13—N2—C14—C15	69.63 (12)
C14—N2—C7—C6	11.99 (15)	C7—N2—C14—C15	−121.05 (11)
C1—C6—C7—N1	−152.39 (9)	C19—C14—C15—C16	0.04 (14)
C5—C6—C7—N1	23.89 (13)	N2—C14—C15—C16	−176.77 (8)
C1—C6—C7—N2	24.60 (14)	C14—C15—C16—C17	0.01 (15)
C5—C6—C7—N2	−159.12 (9)	C15—C16—C17—C18	−0.62 (15)
C7—N1—C8—C9	179.70 (10)	C16—C17—C18—C19	1.17 (15)
C7—N1—C8—C13	−0.46 (10)	C15—C14—C19—C18	0.50 (14)
N1—C8—C9—C10	179.19 (10)	N2—C14—C19—C18	177.29 (9)
C13—C8—C9—C10	−0.64 (14)	C17—C18—C19—C14	−1.10 (15)
C8—C9—C10—C11	−0.21 (15)		
